# Biocompatible Nanocomposites for Postoperative Adhesion: A State-of-the-Art Review

**DOI:** 10.3390/nano14010004

**Published:** 2023-12-19

**Authors:** Saeid Kargozar, Sara Gorgani, Simin Nazarnezhad, Andrew Z. Wang

**Affiliations:** 1 Department of Radiation Oncology, UT Southwestern Medical Center, Dallas, TX 75390, USA; saeid.kargozar@utsouthwestern.edu; 2 Tissue Engineering Research Group (TERG), Department of Anatomy and Cell Biology, School of Medicine, Mashhad University of Medical Sciences, Mashhad 917794-8564, Iran; gorganis991@mums.ac.ir (S.G.); nazarnezhads@mums.ac.ir (S.N.)

**Keywords:** postoperative adhesion, antiadhesive agents, biopolymers, biocompatible nanocomposites, drug delivery, tissue healing, tissue engineering

## Abstract

To reduce and prevent postsurgical adhesions, a variety of scientific approaches have been suggested and applied. This includes the use of advanced therapies like tissue-engineered (TE) biomaterials and scaffolds. Currently, biocompatible antiadhesive constructs play a pivotal role in managing postoperative adhesions and several biopolymer-based products, namely hyaluronic acid (HA) and polyethylene glycol (PEG), are available on the market in different forms (e.g., sprays, hydrogels). TE polymeric constructs are usually associated with critical limitations like poor biocompatibility and mechanical properties. Hence, biocompatible nanocomposites have emerged as an advanced therapy for postoperative adhesion treatment, with hydrogels and electrospun nanofibers among the most utilized antiadhesive nanocomposites for in vitro and in vivo experiments. Recent studies have revealed that nanocomposites can be engineered to generate smart three-dimensional (3D) scaffolds that can respond to different stimuli, such as pH changes. Additionally, nanocomposites can act as multifunctional materials for the prevention of adhesions and bacterial infections, as well as tissue healing acceleration. Still, more research is needed to reveal the clinical potential of nanocomposite constructs and the possible success of nanocomposite-based products in the biomedical market.

## 1. Introduction

Postoperative adhesion represents one of open surgery’s most common and challenging complications [1]. Post-surgery adhesions can occur anywhere on the body that has undergone an open manipulation, including the abdominopelvic, gynecologic, cardiovascular, and nervous system [2,3,4,5]. However, complications, such as chronic infection and endometriosis, may also lead to tissue adhesion [6]. The morbidity and incidence of postoperative adhesions are higher in abdominopelvic areas (peritoneal cavity) due to the peritoneum’s unique histological characteristics, as well as the multi-organ nature of the peritoneal cavity [7]. To illustrate this, a ten-year prospective research study carried out on 12,584 patients undergoing abdominal surgeries revealed a high prevalence (93%) of adhesion-related complications [8,9]. The high rate of postoperative adhesions and adhesion-related complications not only resulted in a high economic burden on patients and the healthcare system, but also resulted in serious long-term health problems for patients. From a morphological perspective, adhesions represent undigested, non-anatomical fibrous tissue connections (rich in fibroblasts) between the surgery site and adjacent tissues and organs (e.g., intestines) or the abdominopelvic wall [10]. Adhesion symptoms usually present as chronic pain (e.g., abdominal and pelvic pains) and may be followed by severe life-threatening conditions like small bowel obstructions [11]. Secondary infertility in females, organ movement limitation, and dysfunction are other complications associated with postsurgical adhesions [12,13]. Adhesions can be classified as de novo (type I) and secondary (type II), according to their development site and formation cause (surgery or not) [14]. De novo adhesion refers to adhesions that are formed in the body after surgery regardless of their location, whereas secondary adhesions result from either previously performed surgery or complications other than previous surgery (e.g., infections) [10,15]. Three core biological events happen during postoperative adhesion formation: (I) the inhibition of the fibrinolytic system and extracellular matrix (ECM) degradation, (II) induction of an inflammatory reaction, and (III) induction of tissue hypoxia and subsequent promotion of angiogenesis [16].

Up to now, a wide range of approaches have been proposed and employed to reduce and prevent postoperative adhesions, including improved surgical procedures, the use of specific pharmaceutical drugs (anti-inflammatory, anticoagulation, and anti-fibrosis medications), and combined therapies. However, the use of tissue-engineered (TE) constructs (biomaterials and three-dimensional (3D) scaffolds) has gradually become common practice for postoperative adhesion management in the era of modern medicine. In this regard, various biomaterial-based antiadhesive products have been commercialized and clinically used in different forms, including powders, solutions, sprays, injectable hydrogels, sponges, and films [17]. Most antiadhesive products are indeed biopolymer-based constructs and suffer from some inherent limitations, such as poor biocompatibility and mechanical properties [18,19,20]. It is well documented that natural polymers lack sufficient processability, brittleness, and tensile strength, while also undergoing rapid degradation. Using synthetic polymers, however, could remove these obstacles to some extent, but there are still some concerns regarding their biocompatibility like potential toxic effects, inflammation, and so on. To overcome these limitations, it is commonly suggested to fabricate composites by combining both natural and synthetic polymers to enhance their physicomechanical properties [21]. Recently, nanocomposites have received much attention for their use in treating postsurgical adhesions due to their superior physicochemical, mechanical, and biological features (e.g., enhanced biocompatibility and mechanical properties), as compared to their polymeric counterparts [22]. Current research indicates that there are excellent opportunities in utilizing antiadhesive nanocomposites for the treatment of postoperative adhesions, along with managing other complications (e.g., tumor recurrence) [23]. In this regard, nanocomposites can easily be engineered as multifunctional, stimuli-responsive constructs that can facilitate tissue healing acceleration through hemostasis maintenance, microbial infection inhibition, and cell growth stimulation and proliferation [24].

Most developed and employed nanocomposites are indeed organic–organic composites; thus, it may be interesting to explore the usability of organic–inorganic nanocomposites in managing postoperative tissue adhesions. It is worth noting that inorganic materials, including clays, carbon-based nanomaterials, metal oxides (e.g., bioceramics and bioactive glasses), and metallic nanoparticles, can reinforce the mechanical properties, anti-microbial performance, and cellular behavior of antiadhesive nanocomposites [25,26]. For instance, gold nanoparticles demonstrated lower microscopic and macroscopic peritoneal adhesion scores when compared with the untreated group in a rat animal model [27]. Additionally, taking advantage of novel technologies (e.g., 3D printing and customized additive manufacturing) in preparing TE constructs plays a substantial role in the next generation of antiadhesive substances. This paper is a comprehensive review that aims to introduce and criticize various types of antiadhesive biomaterials and scaffolds with a focus on the critical significance of biocompatible nanocomposites in the current and future fields. Accordingly, this work may be helpful for researchers and scientists who are trying to develop and introduce the next generation of antiadhesive products for possible clinical use.

## 2. Pathophysiology of Adhesions

**Figure 1:**
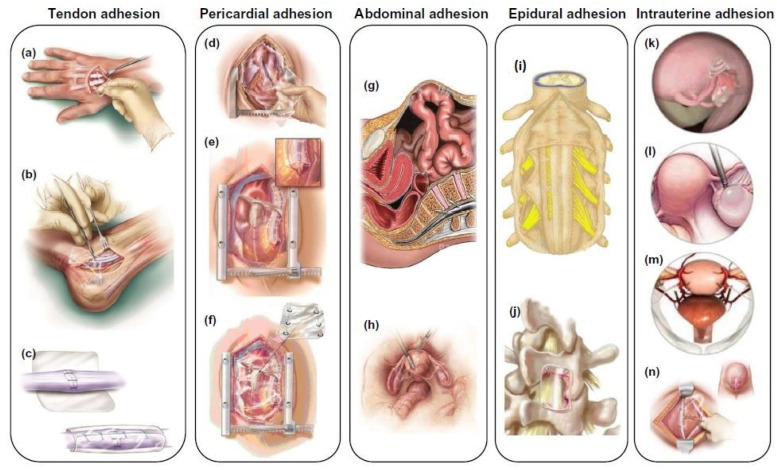
Schematic illustration of different types of tissue adhesion, including tendon adhesion (**a**–**c**), pericardial adhesion (**d**–**f**), abdominal adhesion (**g**,**h**), epidural adhesion (**i**,**j**), and intrauterine adhesion (**k**–**n**). Reproduced with permission from [21].

Internal cavities of the body (e.g., thoracic and abdominopelvic cavities) are covered by a thin monolayer of loosely connected mesothelial cells, also known as the mesothelium, which is uniquely named for each cavity (pleura/peritoneum). The loose connection of monolayer cells and their delicacy have made mesothelial coverings susceptible not only to invasive traumas (e.g., surgery), but also to stressful conditions (e.g., exposure to talc powder of gloves, infection) [13]. Any disruption in mesothelial coverage can expose the basement membrane and underlying connective tissue to other adjacent tissues, which can trigger local inflammation. In the case of laparoscopies and laparotomies, postoperative adhesion can occur at the surgery site and is characterized as the initial and normal response of the body that deals with induced damage (Figure 1). In fact, the body begins the repair process of the injured tissue through the activation of coagulation, inflammation, proliferation, and remodeling pathways (i.e., the normal wound-healing process). In the case of adhesions, an imbalance between fibrin deposition and fibrinolysis arises during the wound-healing process, which leads to downregulated fibrinolysis and, consequently, adhesion development [25]. Providing precise information regarding affected tissues, including their repair process and the mechanisms by which adhesion formation develops, can help in discovering possible effective solutions to reduce and inhibit postoperative adhesions.

Connective tissue is a rich pool of various cells, including fibroblasts, macrophages, and mast cells. These cells are pioneers in the inflammation cascade, once the peritoneum is damaged [26]. Mesothelial cells secrete peritoneal fluid, which is a rich source of blood histocytes, macrophages, mast cells, erythrocytes, and small amounts of mesothelial cells. The primary function of peritoneal fluid is lubricating and disinfecting the abdominopelvic cavity [27]. Importantly, mesothelial cells can gain profibrotic phenotypes, also known as adhesion phenotypes or myofibroblasts, when exposed to pro-inflammatory factors and undergo a mesothelial-mesenchyme transition (MMT). The MMT enables mesothelial cells to secrete inflammatory factors, chemokines, growth factors, and extracellular matrix (ECM) elements and facilitates the healing process [28]. In the following section, we will describe the underlying mechanisms related to different stages of postoperative adhesion, such as coagulation, inflammation, and fibrinolysis. These phases commonly occur in a parallel and overlapping time frame, in which the activation of each phase can potentially activate others.

### 2.1. Coagulation and Inflammation

**Figure 2:**
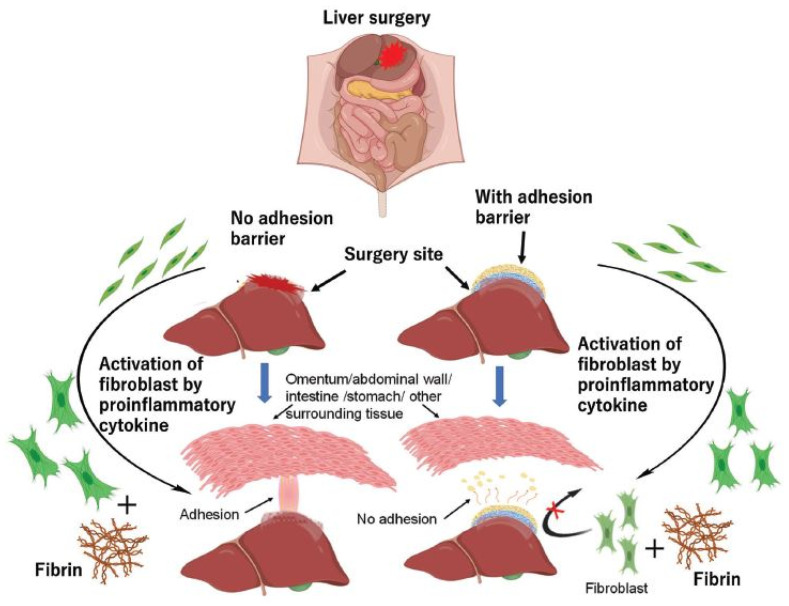
A schematic representation of the role of fibrin and fibroblasts in the formation and inhibition of tissue adhesion. Reproduced with permission from [47].

Once the peritoneum integrity is disrupted, hemostasis and inflammation cascades are overlapping processes that suppress bleeding and prevent potential infections in the damaged areas. The local release of histamine and other signaling molecules (i.e., cytokines and growth factors) stimulates blood vessels for increased permeability, leading to the promoted recruitment and infiltration of inflammatory cells, specifically neutrophils and macrophages, around the wound area. Following local blood vessel injury, the endothelial cell monolayer can be interrupted, leading to von Willebrand factor (VWF) and tissue factor (TF) exposure, both of which exist in the vessel walls. VWF binds to platelets and mediates interactions between platelets and exposed collagen, resulting in platelet aggregation and platelet plug generation. With the initiation of coagulation, fibrinogen in the plasma is exuded, and many prothrombins are activated into thrombin due to platelet-released factors [29,30]. In the meantime, fibrinogen molecules are converted to fibrin through thrombin, consequently coagulating with aggregated platelets to generate a stable clot. Subsequently, the released cytokines from platelets, as well as the products resulting from the clot’s degradation, recruit more macrophages, neutrophils, mast cells, T cells, and mesothelial cells [31,32]. In fact, the fibrin deposition over the injured area can prepare a provisional matrix that facilitates inflammatory cell migration and subsequent inflammation (Figure 2). Immediately after the peritoneal injury, a huge number of inflammatory cells, mainly lymphocytes, monocytes, and neutrophils, infiltrate along with the peritoneal fluid. These cells can secrete various cytokines, including interleukin-1β (IL-1β), IL-6, tumor necrosis factor α (TNF-α), and vascular endothelial growth factor-A (VEGF-A), which are directly associated with the degree of adhesion [32]. IL-1, IL-6, and TNF-α may also be secreted from macrophages that are differentiated from peritoneal fluid monocytes and mesenteric mesenchymal stem cells [33,34,35,36]. Also, peritoneal macrophages may secrete tissue plasminogen activator (tPA), which is involved in the fibrinolysis process. As a matter of fact, there are two macrophage phenotypes, M1 (classical macrophages) and M2 (alternatively activated macrophages). M1 can secrete different cytokines (e.g., IL-1α, IL-1β, and TNF-α) and lead to the elimination of microorganisms, activation of nitric oxide synthase (NOS), and subsequent tissue damage in the case of excessive oxidative stress. In contrast, M2 macrophages have an anti-inflammatory role in the late stages of inflammation and contribute to wound healing and fibrosis. Pro-inflammatory cytokines released from M1 macrophages can bind to the surface receptors of mesothelial cells and activate the NF-κB signaling cascade, which consequently promotes the secretion of subsequent inflammatory molecules. Simultaneously, the activated Rho signaling pathway may cause the overexpression of plasminogen activator inhibitor (PAI-1), and hence suppress the peritoneal fibrinolysis system’s performance [28]. Activated neutrophils and macrophages can lead to increased oxidative stress within the mesothelium and promote adhesion formation [37]. Furthermore, the subsequent extra hydrogen peroxide and superoxide anions generated are cytotoxic and can damage cells, e.g., endothelial cells, platelets, and fibroblasts [35]. Cytolysis and lipid peroxidation of cell membranes can result in enhanced vascular permeability and further exudate leakage, facilitating and expediting the adhesion process. Moreover, hyaluronic acid (HA) secretion from the peritoneal mesothelium can act as a recruitment factor for inflammatory and immune cells, promoting the expression of transforming growth factor-β (TGF-β) and TNF-α [38], which play substantial roles in fibrosis and adhesion development. Several cellular pathways, along with the TGF-β1 signaling pathway, can contribute to fibrosis. These pathways (e.g., PI3K/AKT, Wnt/β-catenin) can proceed with the irreversible conversion of the mesothelium to myofibroblasts [39,40,41]. Myofibroblasts can form adhesions in the peritoneal cavity due to their high-level expression of collagen (type I and III), matrix metalloproteinase-1 (MMP-1), tissue inhibitor of metalloproteinase-1 (TIMP-1), VEGF, and other factors [42,43]. C-X-C motif ligand 1 (CXCL1) and monocyte chemoattractant protein 1 (MCP-1) produced by recruited neutrophils and monocytes can lead to adhesions through the activation of angiogenesis and fibrin deposition [44,45,46]. In conclusion, the inflammation phase can regulate adhesion development through the recruitment of cells and the secretion of chemokines. The final products of this step can stimulate further fibrin and ECM deposition.

### 2.2. Fibrinolysis

Fibrin serves as a temporary matrix during tissue repair and is removed after the healing process. The appropriate performance of the fibrinolytic system (anticoagulation system), alongside the inflammation and coagulation processes, is required to dissolve the fibrin scaffold. When fibrin does not degrade in the desired time frame, fibroblasts synthesize collagen within the fibrin matrix, leading to permanent adhesion. Hence, it is necessary to dissolve extra fibrin in time. The ideal time for adhesion prevention is five to seven days after injury, except for those affected by infection or foreign-body invasion. The fibrinolytic system’s activation is regulated by intact mesothelial cells and macrophages. The final product of this system is plasmin, the activated form of plasminogen. Plasmin can destroy fibrin clots by breaking down the peptide bonds between arginine and lysine in fibrinogen and fibrin, making them soluble substances [48]. Plasminogen–plasmin conversion is controlled by several factors, including tissue/urokinase plasminogen activators (tPA, uPA), plasminogen activator inhibitors (PAI-1, PAI-2), plasmin inhibitors (PIs), and a series of enzymatic reactions. Moreover, thrombin-activatable fibrinolysis inhibitor (TAFI) and α2-antiplasmin (α2-AP) can inhibit this conversion through the inactivation of plasmin [26]. The tPA and uPA activate the inactive precursor plasminogen into plasmin, while PAI-1 can efficiently suppress this activation. The highest amount of tPA is released from vascular endothelial cells (ECs), mesothelial cells, and macrophages [49]. tPA has a strong affinity for fibrin and forms the fibrin–tPA complex, which can rapidly activate plasminogen in blood clots. Plasminogen activation in the abdominal area is mostly (around 95%) regulated by tPA. The binding of uPA to its receptor urokinase-type plasminogen activator receptor (uPAR) can promptly activate plasminogen, which has a significant role in tissue remodeling. Both fibrinogen and fibrin can promote the production of TNF-α and IL-1β, inciting the secretion of PAI-1 from mesothelial and endothelial cells to prevent plasminogen activation. PAI-1 can in turn enhance adipose tissue development, as well as trigger macrophages to increase inflammation in the damaged area, leading to adhesion [50]. It should be noted that there is a synergistic relationship between fibrinolysis and matrix metalloproteinase (MMPs) in wound repair. The main biological task of MMPs is to degrade the ECM. As a major member of the MMP family, MMP-3 can block PAI-1 and α2-AP to increase plasmin amounts. On the other hand, plasmin can activate proMMP-9 to MMP-9 to remodel the ECM [51,52,53]. In addition, normal mesothelial cells can also contribute to fibrinolysis and restrict adhesion formation. Finally, it is worth noting that any disruption in the balance between coagulation and inflammation with fibrinolysis phases can cause adhesion (Figure 2).

### 2.3. Others

In normal conditions the fibrin matrix appears temporarily and is degraded through the fibrinolysis event within three to five days. The fibrinolytic process’s balance is varied based on the operative intervention. In a rat animal model study [54], postoperative adhesion formation was evaluated by measuring the levels of tPA and PAI-1 in three distinct groups: the control nonoperative group, open-surgery group, and laparoscopic surgery group. The collected data revealed that tPA levels were significantly reduced in the laparoscopic operation group versus the nonoperative control and open-surgery groups. Furthermore, the mRNA expression of PAI-1 was higher in the open-surgery group when compared with the laparoscopic group, indicating enhanced adhesion in the open group. These results reflect the minimally invasive nature of laparoscopic surgeries, even when prolonged operations, compared to open surgeries. Indeed, hypoxia-inducible factor-1α (HIF-1α) can bind to the oxygen-sensitive promoter regions of the PAI-1 gene, resulting in its upregulation [55]. It has been reported that the tPA/PAI-1 ratio declined in hypoxic conditions [56,57]. Tissue hypoxia with increased generation of oxygen and nitrogen reactive species, ROS and RNS, respectively, can contribute to enhanced oxidative stress, resulting in DNA damage, cell apoptosis, and the overproduction of oxidized proteins [58]. ROS production has been demonstrated to stimulate adhesion formation by upregulating the expression of several factors, including TNF-α, IL6, type I collagen, and VEGF [59]. Moreover, tissue hypoxia can increase the production of superoxides. It was shown that fibroblasts exposed to superoxides can generate pro-fibrogenic factors, like TGF-β and type I collagen [60]. Additionally, it has been revealed that HIF-1α and TGF-β can both increase the levels of VEGF that are primarily released by mast cells, stimulating vascularization and promoting vascular permeability [61]. Angiogenesis is another physiological phenomenon that contributes to the wound-healing process in response to local ischemia. This process is highly intermingled with inflammation, coagulation, and fibrinolytic processes. VEGF can enhance the deposition of a fibrin-rich ECM to provide a substrate for newly formed vasculature to overcome local ischemia [62]. Furthermore, the angiogenesis process can contribute to the organization and maturation of adhesive bands, and can provide the nourishment of residing cells [63]. Alongside this, fibrinolytic system activity is required to facilitate ECM degradation and then its subsequent remodeling [64]. Regulated parallel activity between the aforementioned systems may reduce adhesion during wound healing; however, this is a rare occurrence, and adhesion takes place as a result of excessive fibrin deposition [25].

Despite all efforts, there is no perfect approach to reducing and preventing postsurgical adhesions. For example, adhesiolysis surgery is usually performed to relieve patients with severe conditions; however, it was reported that 47% of patients need secondary surgery [65]. Preventive practices to inhibit postsurgical adhesions can include (I) improved surgical techniques, (II) utilization of mechanical barriers (e.g., solid membranes and films, as well as fluidic hydrogels), and (III) administration of antiadhesive agents that can interfere with various adhesion processes (e.g., anticoagulant, anti-inflammatory, antioxidant) [48] (Table 1). Consequently, findings have demonstrated that performing minimally invasive surgical approaches, such as laparoscopy, may minimize postsurgical adhesions in candidate patients. However, several ineligible patients return with adhesion complications even after laparoscopic surgery [66]. The burden associated with secondary surgery and postsurgical complications has led to the development and use of barrier patches to prevent adhesions. Up to now, many commercialized antiadhesive biomaterials (e.g., Seprafilm^®^, Spraygel^®^, Adept^®^) have been developed in the form of films, gels, sprays, and solutions, to name a few [50,65,67,68]. However, there are some concerns associated with the use of these barriers, including decreased efficacy attributed to their high biodegradation in the abdominal cavity (liquid barriers), dislocation from the targeted place after surgery, and limited capability to properly cover the affected area [50]. In the following sections, we introduce different types of biocompatible materials utilized for the treatment of postoperative adhesions, and then highlight the beneficial role of nanostructured biocomposites in comparison to commonly used polymeric constructs.

## 3. Biocompatible Materials for Postoperative Adhesions

To date, a wide range of natural and synthetic materials have been processed and developed for managing postoperative adhesions in various locations, such as the peritoneum and heart. Polymers stand out among applied biomaterials in terms of postoperative adhesion prevention due to their biocompatibility and tunable characteristics (e.g., controllable biodegradability and ease of functionalization) [84]. Additionally, polymers can be used for the loading and delivery of a broad range of therapeutic cargoes to improve outcomes. It can be concluded from the literature that theadministration of both natural and synthetic polymers has led to the successful prevention of postsurgical adhesions (Table 2). Although naturally occurring biopolymers exhibit excellent compatibility with living organisms, they suffer from poor mechanical properties, lower productibility, and batch-to-batch variations. Collagen, gelatin, chitosan, alginate, hyaluronic acid (HA), silk fibroin (SF), dextran, chondroitin sulfate, carboxymethyl cellulose (CMC), starch, and cellulose are some of the most commonly used natural polymers for surgical purposes [85]. On the other hand, numerous synthetic biopolymers have been suggested and employed to control postsurgical adhesions. Poly ε-caprolactone (PCL), polyethylene glycol (PEG), polylactic acid (PLA), poly lactic-co-glycolic acid (PLGA), polyvinyl alcohol (PVA), and polyvinylpyrrolidone (PVP) are some examples of well-known synthetic biopolymers utilized for the prevention of postoperative adhesions.

It should be highlighted that many of the mentioned biopolymers are now available as commercialized products in the market for clinical use, including Seprafilm^®^, Sepraspray^TM^, Hyalobarrier^®^, Adept^®^, and SprayGel^TM^ [106] (Table 3). Importantly, each product can be used for specific locations and medical conditions in the body according to the manufacturer’s guidelines. For example, resorbable membranes made of HA and CMC in the form of a film (Seprafilm^®^, Sanofi/Genzyme) or a woven fabric (Interceed^®^, Ethicon) were indicated only for the management of abdominal adhesions [107]. Biopolymer-based products are commonly formulated into diverse forms (e.g., films, hydrogels, solutions, sprays, and porous scaffolds) for use as antiadhesive constructs. In this regard, several products, such as polymer-based films, have been commercialized and are available on the market. For instance, Seprafilm^®^, a mechanical bioresorbable antiadhesive barrier, received FDA market approval in 1996. It is made of HA and CMC, which can be converted to a gel form 24 to 48 hours post-operation and remains in the area for up to seven days. However, research on novel antiadhesive film compositions is in progress, since no significant differences were reported regarding their potential in adhesion prevention [71]. It is important to note that electrospun nanofibers with different compositions were also studied as barrier membranes for antiadhesive strategies. Electrospun nanofibrous scaffolds offer specific advantages for treating postsurgical adhesions, including ease of fabrication and modification, high surface area, and drug-loading capability. Although several electrospun nanofibers were successfully utilized to reduce adhesions, some studies have mentioned that electrospun nanofibers fail to completely prevent adhesion formation. In this regard, polymer degradation may interfere with the antiadhesion process in vivo; for example, PLA degradation was shown to hamper the antiadhesion effectiveness against peritendinous adhesions by promoting M2 macrophage polarization mediated by enhanced STAT6 phosphorylation and activated myofibroblasts [108]. Therefore, optimizing the rate of electrospun nanofiber biodegradation should be considered to reduce possible side effects. In addition, loading therapeutic biomolecules and drugs onto electrospun nanofibers was found to be useful for obtaining satisfying outcomes [109,110].

As naturally derived constructs, decellularized extracellular matrixes have been studied for preventing postsurgical adhesions and promoting tissue repair and regeneration [111,112,113]. To exemplify this, a decellularized tendon matrix (DTM) has been successfully prepared through the decellularization of bovine tendon tissue and employed as an antiadhesive construct [114]. The results showed that DTM could degrade after subcutaneous implantation over 12 weeks without any significant signs of inflammatory reaction. Furthermore, this natural construct could prevent tendon adhesion in the Achilles tendon of rabbits, along with accelerated tendon repair and regeneration.

Injectable hydrogels are considered the most promising tissue-engineered construct in the prevention of postoperative tissue adhesions [107,115]. Hydrogels exhibit outstanding characteristics for use in postsurgical adhesions, including antifouling capability and the possibility of loading and controlled release of different drugs at desired sites. Moreover, hydrogel crosslinking can be performed by using various physical (e.g., interaction between ions, hydrogen bonding) or chemical (e.g., photo-initiated hydrogels, enzymatic reactions, “Click” reactions) approaches to modulate and customize their properties [115]. Stimuli-responsive hydrogels are a specific class of hydrogels that undergo structural, mechanical, and biochemical changes in response to environmental cues (e.g., heat). They are known as useful constructs in drug delivery and tissue-engineering applications [116]. Concerning antiadhesive applications, thermo-responsive hydrogels composed of poly(N-isopropyl acrylamide) (PNIPAM) grafted with chitosan (CS) and hyaluronic acid (HA) were shown to have superior capabilities in preventing postoperative paratendinous adhesion. These hydrogels exist in a free-flowing form before injection, and are then converted to a gel in the body, leading to the separation of the wound surface from adjacent tissues and organs with negligible invasiveness [117]. In some studies, drug-loaded hydrogels were investigated for managing postoperative adhesions in vivo. The obtained data from these studies has been promising in terms of preventing adhesion formation through pharmacological processes [118]. For example, the administration of naproxen-nanoparticle-containing chitosan hydrogels in a rat model of abdominal cecum adhesion led to the prevention of postoperative abdominal adhesions and pain relief without any significant adverse effects to vital organs, including the liver, spleen, heart, lung, and kidneys [119]. Optimal dosage selection and the sustained release of loaded drugs are mentioned among the most challenging issues for the extensive usage of drug-loaded hydrogels.

Recently, porous scaffolds based on droplet microfluidics have attracted huge research interest for the prevention of specific postsurgical adhesion cases, such as intrauterine adhesions. Notably, Cai et al. utilized microfluidics for the production of monodisperse droplet templates of gelatin methacryloyl (GelMA) and sodium alginate (Na-alginate) and then fabricated 3D scaffolds having an external-internal connected pore structure with the assistance of ultraviolet (UV) and calcium chloride solidification [120]. They first investigated different aspects of the produced scaffolds (e.g., biocompatibility, compressibility, swellability, degradation rate, and drug release profile) in vitro. Next, bFGF-loaded scaffolds were transplanted into an IUA rat model to determine their potential in the prevention of postoperative adhesions. The authors observed excellent biocompatibility and biodegradability of the fabricated scaffolds, along with proper compressibility for delivery to the uterus through the vagina. Moreover, the scaffolds regenerated the damaged endometrium in the IUA rat model, providing proof of their value in preventing IUAs. It should be noted that the potential of stem-cell-loaded constructs has been evaluated for preventing tissue adhesions [41,121]. For example, bone-marrow-derived mesenchymal stem cells (BMSCs) were loaded onto scaffolds made of poly (glycerol sebacate) (PGS), a poly(lactic-co-glycolic acid) (PLGA) scaffold, and collagen and subsequently transplanted into a wounded rat uterus model. The reported findings indicate that the administration of BMSC-loaded PGS constructs can result in better regeneration (thicker endometrium with more glands) of the damaged uterus and prevent intrauterine adhesions (IUAs) compared to the other groups. This construct was introduced as a promising alternative to estrogen-containing intrauterine devices that are clinically used for preventing intrauterine adhesions (IUAs) and promoting endometrium regeneration after surgical synechiotomy.

## 4. Biocompatible Nanocomposites: New Players in Postoperative Adhesions

**Figure 3:**
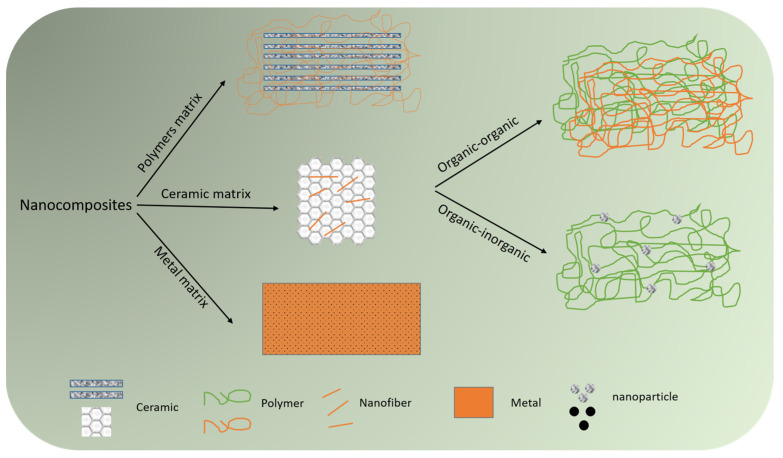
Classification of nanocomposites based on constituent matrix (polymer, ceramic, or metal) and incorporated phases.

Compared to bulk materials, the use of nanosized materials (nanomaterials) has led to substantial improvements in biomedical strategies due to their unique characteristics, such as higher surface area and reactivity [122]. Nanocomposites are heterogeneous materials that are composed of matrixes and nanosized fillers. Depending on the incorporated matrix material, they are divided into ceramic matrix nanocomposites (CMNC), polymer matrix nanocomposites (PMNC), and metal matrix nanocomposites (MMNC). The integrated nanosized filler phase can be in any shape (nanofiber, nanoparticle, nanosheet, etc.) and from an organic/inorganic source. Therefore, depending on the contributing phases, nanocomposites can be organic-organic or organic-inorganic [123] (Figure 3). The incorporation of nanoparticles into polymeric matrixes results in biocompatible nanocomposite generation that can be utilized for diverse biomedical applications, such as treating postoperative adhesions. Both naturally occurring substances (e.g., amnion) and synthetic biomaterials (e.g., PLA) have been utilized as polymeric substrates of biocomposites [124,125]. Nanocomposites offer exceptional benefits for treating postoperative adhesions, including improved biocompatibility and enhanced mechanical properties, which can be achieved through various methods, including crosslinking, hybridization/reinforcement with nanoparticles, etc. [126,127,128]. Moreover, they benefit from tunable and controlled biodegradability, which means that various compositions of natural- and/or synthetic-based biomaterials can be designed to generate a biocompatible nanocomposite possessing a controlled degradation rate synchronized with tissue repair [17]. Given adhesion’s role in infectious disease development, it is worth mentioning that antiadhesive nanocomposites can inhibit the adhesion of microorganisms. This can lead to the development of a new generation of anti-microbial nanocomposites for several strains that are resistant to common antibiotics [129]. Based on the literature, it can be stated that hydrogels and electrospun nanofibers form the main types of antiadhesive nanocomposites [130,131,132].

Polysaccharide-based composite hydrogels made of N, O-carboxymethyl chitosan (N, O-CS) and oxidized dextran (ODA) were previously developed as self-healing injectable antiadhesion barriers with antibacterial and hemostatic capacities [133]. In fact, carboxymethyl was added to the N and O positions of glucosamine and N-acetylglucosamine units of chitosan to improve its solubility under physiological conditions. On the other hand, the crosslinking of the ODA aldehyde functional group with the N, O-CS amino functional group modulated by the Schiff base resolved the need for any toxic crosslinking agents or radiation sources. Accordingly, the fabricated hydrogels showed excellent biocompatibility and hemocompatibility in vitro and in vivo with an optimal degradation rate. After 14 days of surgery, these hydrogels could inhibit the adhesion of fibroblasts to the injured abdominal wall in rats, thereby preventing tissue adhesion formation even better than commercial carboxymethyl chitosan hydrogels. Recent progress in the field has led to the introduction of a new generation of hydrogels that show unique characteristics for postoperative adhesions as compared to conventional hydrogels (covalently crosslinked hydrogels). In this regard, dynamically crosslinked supramolecular polymer–nanoparticle hydrogels were produced from hydrophobically modified HPMC-C12 and PEG-PLA nanoparticles as an effective postoperative pericardial adhesion barrier [107]. The hydrogels were shear-thinning and self-healing with viscoelastic flow properties that could be sprayed with standard equipment. The results from the in vivo study performed on a rat model with severe pericardial adhesions indicated that the hydrogels could adhere to tissue due to their adhesive part (HPMC-C12), as well as reduce the severity of pericardial adhesions even better than two commercial products, Seprafilm^®^ (film) and Interceed^®^ (fabric). Moreover, the fabricated hydrogel nanocomposites could successfully decrease the severity of cardiac adhesions in a cardiopulmonary-bypass model in sheep compared to untreated animals.

**Figure 4:**
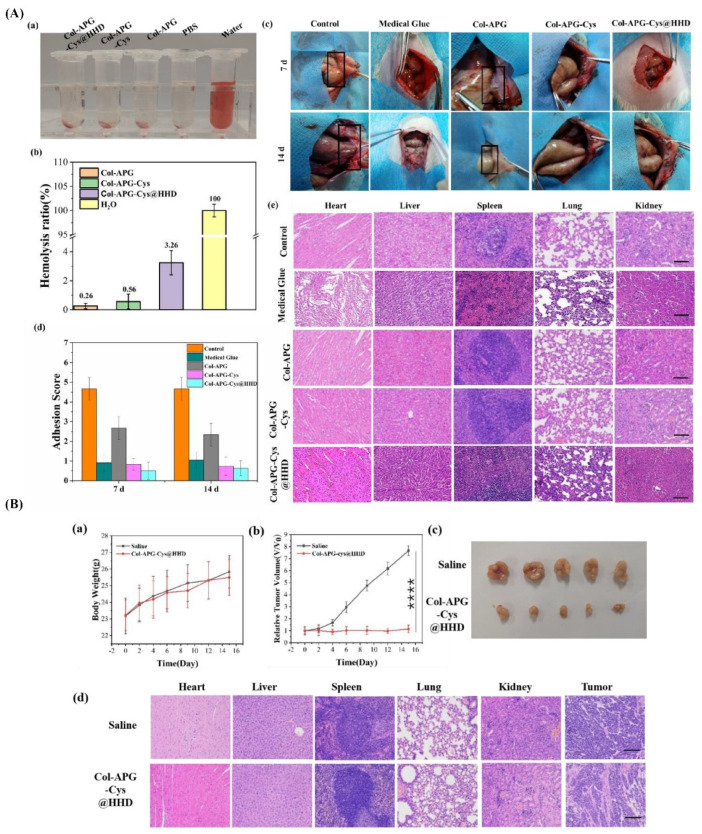
Antiadhesive and anti-cancer properties of COL-APG_Cys@HHD hydrogel. (**A**) (**a**) Optical image of the hemolysis interaction between blood and five distinct groups, namely, Col-APG-Cys@HHD, Col-APG-Cys, Col-APG, PBS, and water. (**b**) Quantitative representation of the hemolysis ratio of relevant samples. (**c**) Macroscopic observation of the control group and the antiadhesive effect of medical glue, Col-APG, Col-APG-Cys, and Col-APG-Cys@HHD hydrogels on tissue. (**d**) Distribution of adhesion scores on days 7 and 14 after surgery in the corresponding groups. (**e**) Histopathological evaluation of normal organ sections collected from animals treated with saline, medical glue, Col-APG, Col-APG-Cys, and Col-APG-Cys@HHD hydrogels after sacrifice through H&E staining. The scale bar shows 100 µm. (**B**). (**a**) Variations in body weight of tumor-bearing mice for the duration of therapy. Data are shown as mean ± SD, n = 5. (**b**) Tumor growth curves after tail IV injection of saline and Col-APG-Cys@HHD. Data are shown as mean ± SD, n = 5. (**** *p* < 0.0001) (**c**) Macroscopic images of the tumor size stripped from mice in control and Col-APG-Cys@HHD groups. (**d**) H&E staining of normal organs and tumor sections collected from animals treated with saline and Col-APG-Cys@HHD at the end of the experiment. The scale bar shows 100 µm. Reproduced with permission from [23].

Recently, the development and utilization of multifunctional composite hydrogels have become a trend for the simultaneous prevention of postoperative adhesions and tumor recurrence [134]. For example, pH-responsive nanocomposite hydrogels were prepared using collagen (Col) and recombinant albumin nanoparticles (HHD NPs) crosslinked with aldehydeylated polyethylene glycol (APG6K) as a potential construct for overcoming two major problems in the postoperative treatment of abdominal tumors (i.e., abdominal adhesion and tumor recurrence [23] (see Figure 4)). One side of the hydrogel surface was coated with zwitterionic cysteine (Cys) to form a hydration layer that hinders protein and cell attachment, subsequently reducing adhesion between tissues. The other side of the construct served as an adhesive surface due to the presence of collagen. This hydrogel system released HHD NPs under acidic conditions, which is commonly observed in the cancer microenvironment. The released NPs led to a sharp decrease in the survival rate of cancer cells (HeLa cell line) compared to normal cells (NIH 3T3 cell line). The results of this in vivo study showed that hydrogels could prevent intraperitoneal adhesion in the lateral wall defect of a cecal abrasion rat model. This drug-loaded system could inhibit tumor growth in female Balb/c-nu nude mice, as the tumor’s volume was eight times smaller than that in the control group on the fifteenth day, with minimal side effects on the animals’ organs (e.g., heart and liver).

Electrospun nanofibrous nanocomposites have shown great promise as barrier membranes in postsurgical adhesion management [44,135,136,137]. However, commercially available membranes suffer from some restrictions; for example, Seprafilm^TM^ and Interceed^TM^ are considered to have poor mechanical properties and performance with blood contact, respectively. Accordingly, researchers have made huge efforts to develop novel formulations of electrospun mats with improved mechanical and biological features. In this regard, gelatin and CMC were previously added to PCL to fabricate tricomposite nanofiber barrier membranes with improved hydrophilicity, mechanical stability, and biocompatibility [138]. Another crucial issue in using electrospun mats is associated with their surface characteristics, which commonly provide a suitable surface for cell attachment and growth. This can be a critical challenge once adjacent tissue cells attach and grow on the membrane, resulting in severe tissue adhesion. Therefore, electrospun membrane surface modification has been suggested as a reasonable approach to improve their performance in vivo. In this regard, the surface of electrospun nanocomposites made of PLA/photo-initiator Irgacure-2959 underwent nanoscale coating to generate a super-lubricated nano-skin (SLNS) on the surface of nanofibers [139]. The surface-modified constructs exhibited ideal tensile properties and biocompatibility, and in vivo studies in rat tendon and abdominal adhesion models supported their acceptable antiadhesive performance.

Membrane nanocomposites fabricated by coaxial electrospinning techniques are generally known as appropriate vehicles for the delivery of various therapeutic cargoes to desired locations. Concerning postsurgical adhesions, a series of core-shell nanofibrous membranes were prepared in which HA/platelet-rich plasma (PRP) and PCL serve as the core and shell of electrospun nanocomposites, respectively [140]. PRP is a rich source of bioactive molecules (e.g., growth factors TGF-β1, PDGF, FGF-2) that can help accelerate wound healing (e.g., tendon recovery after tendon injury). The shell of the nanocomposites was prepared by solving PCL in ethylene chloride and N, N′-dimethylformamide (volume ratio = 4:1) to obtain a PCL polymer solution (8%(*w*/*v*)). The optimized core solution had a composition of 1.75% (*w*/*w*) PRP, 1.75% (*w*/*w*) HA, and 0.5% PEO at a 1/1 HA/PRP mass ratio. The PRP-loaded membrane scaffolds prevented penetration and decreased the attachment/focal adhesion of NIH/3T3 mouse embryonic fibroblasts while promoting tenocyte migration in vitro. In addition, the implantation of drug-loaded nanocomposites reduced tendon adhesion formation and inflammation and improved the healing process in the rabbit flexor tendon rupture model. The use of organic-inorganic nanocomposites made of polymers and nanoparticles offers another interesting class of constructs for treating postsurgical adhesions. On this matter, core-sheath nanofiber membranes were previously prepared by embedding silver nanoparticles (Ag NPs) in a PLA nanofiber sheath, with HA in the nanofiber core, and used to prevent postoperative tendon adhesions in peritendinous antiadhesion rabbit models [134]. The thicknesses measured for a thick sheath (Tk) and thin sheath (Tn) without Ag NPs and a thick sheath (Tk+) and thin sheath (Tn+) containing Ag NPs were 643 ± 119, 680 ± 167, 692 ± 165, and 725 ± 226 nm, respectively. The in vitro experiments demonstrated that nanocomposites with thin sheaths containing Ag NPs possess suitable antibacterial activity and could prevent fibroblast penetration and attachment with negligible cytotoxicity. Moreover, in vivo data indicate that this nanocomposite can meaningfully prevent peritendinous adhesions and moderate inflammatory reactions in comparison to its thin-sheath counterparts and the PLA-based commercial adhesion barrier film (SurgiWrap^®^). Another interesting class of nanocomposites used to prevent postoperative adhesion includes multilayer membranes made of electrospun nanofibers and naturally occurring substances. In this regard, electrospun PCL-amniotic membrane composites were successfully developed and evaluated for preventing postsurgical tendon adhesions [141]. For instance, fresh amnions were first freeze- and vacuum-dried, with both sides then coated with PCL nanofibers using electrospinning techniques. In fact, PCL nanofibers were assumed to act as the outer layer and mimic the function of a natural tendon sheath to prevent the invasion of fibroblasts and other tissues. The amniotic membrane was assumed to serve as the intermediate layer for promoting the tendon’s endogenous healing via the release of bioactive molecules (e.g., TGF-β1, bFGF, PDGF, and VEGF). Subsequently, diffusion of the aforementioned molecules from nanofiber pores into the injured site can lead to improved tenocyte proliferation and collagen synthesis. The in vitro assessments of this bioactive-molecule-enriched nanocomposite showed that the upregulation of the phosphorylation of ERK1/2 and SMAD2/3 resulted in improved tenocyte proliferation and enhanced collagen synthesis. Furthermore, in vivo administration of the nanocomposite in the rabbit tendon repair model confirmed that this system can effectively inhibit exogenous adhesion and enhance endogenous tendon healing.

**Figure 5:**
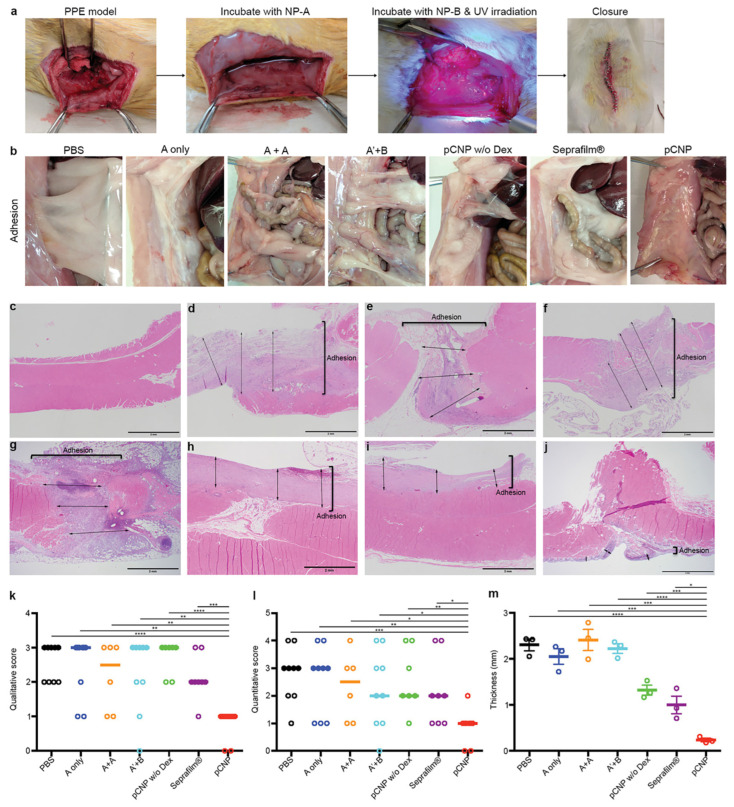
Photo-crosslinkable nanopatch (pCNP) prevents postoperative peritoneal adhesion in a parietal peritoneal excision (PPE) model in rats. (**a**) Macroscopic images representing PPE and the administration of pCNP. (**b**) Representative images of postsurgical adhesions in rats after 14 days of treatment with PBS (the injured site was incubated with saline followed by UV irradiation); A only (the injured site was incubated with NP-A followed by saline under UV irradiation); A + A (the injured site was incubated with NP-A followed by NP-A again under UV irradiation); A′ + B (the injured site was incubated with NP-A′ (NP-A without targeting ligand) followed by NP-B under UV irradiation); pCNP w/o Dex (the injured site was incubated with NP-A without dexamethasone 21-palmitate followed by NP-B under UV irradiation); Seprafilm^®^ (the injured site was incubated with saline under UV irradiation, the saline was wiped out, and the injured site was covered with Seprafilm^®^); and pCNP (the injured area was incubated with NP-A followed by NP-B under UV irradiation). (All the incubation and irradiation times were 10 min.) (**c**–**j**) H&E staining photographs representing the thickness of adhesion/fibrosis in untreated animals (**c**) and rats that underwent surgery and were further treated with PBS (**d**), NP-A only (**e**), NP-A + NP-A (**f**), NP-A′ + NP-B (**g**), pCNP without dexamethasone 21-palmitate (**h**), Seprafilm^®^ (**i**), and pCNP (**j**). The scale bar shows 2 mm. (**k**,**l**) Qualitative (**k**) and quantitative (**l**) scoring analysis of postoperative adhesions in rats 14 days after treatment. Data presented as scatter dot plot with median line (for A + A, n = 6; for pCNP w/o Dex, n = 7; for Seprafilm^®^, n = 8; for other groups, n = 9). (**m**) Quantitative evaluation of the adhesion/fibrosis thickness in (**d**–**j**). Data are shown as mean ± standard error of the mean (SEM), n = 3. * *p* < 0.05, ** *p* < 0.01, *** *p* < 0.001, **** *p* < 0.0001. Reproduced with permission from [143].

In addition to injectable hydrogels and electrospun nanofibrous membranes, other forms of nanocomposites were also developed and employed as antiadhesive barriers; for example, 3D PCL scaffolds were fabricated using a salt-leaching technique and loaded with biphasic calcium phosphate and silver nitrate to serve as bioactive and bioresorbable as well as antibacterial composites, respectively. The obtained data revealed that the silver-functionalized scaffold could significantly decrease the adhesion and growth of staphylococci on the biocomposite. Moreover, polydopamine (PDA)-human keratinocyte growth factor (KGF) nanoparticles (PDA-KGF NPs) combined with hyaluronate (HA) have been examined for preventing postoperative abdominal adhesion formation in rats [142]. The published results indicated that nanoparticles (150 to 210 nm) could not only effectively prevent the incidence of abdominal adhesions in the receiving animals, but also improved mesothelial cell repair in the injured peritoneum. This nanocomposite could decrease collagen deposition and fibrosis in the injured peritoneum and inhibit inflammatory reactions. In another study, Andrew Z. Wang’s research group developed a photo-crosslinkable nanopatch and studied its effectiveness in a rat parietal peritoneum excision (PPE) model [143]. The nanopatch was composed of two nanoparticles: PEG-PLGA functionalized with a collagen IV-targeting peptide (Col-PEG-PLGA) (namely, NP-A) and PLGA-PEG covered with a branched polyethyleneimine (PEI) shell (namely, NP-B). They encapsulated dexamethasone 21-Palmitate (Dex-Pal) with negatively charged NA-A at a concentration of 65.2 ± 4.02 µg mg^−1^ to prevent adhesion formation. Positively charged NP-B was surface-functionalized with diazirine groups to provide photo-induced crosslinking among the nanoparticles. NP-A was administered to the injured site to investigate its potential for specific binding to the basement membrane, which is exposed following mesothelial damage. Then, an NP-B-containing suspension was administered to the damaged site to rapidly form a dense layer mediated by ionic adsorption between the oppositely charged nanoparticles. Finally, the injury site was exposed to UV light at a 365 nm wavelength to initiate the crosslinking process between the two nanoparticles, forming a nanopatch as a specific and dense biological barrier between the injured peritoneal surfaces. After two weeks of treatment, histological observations confirmed the improved performance of the developed nanopatch in preventing postsurgical peritoneal adhesions compared to other groups (phosphate-buffered saline (PBS), only NP-A, NP-A without targeting ligand and NP-B, NP-A without dexamethasone 21-palmitate, and Seprafilm^®^) (Figure 5).

## 5. Conclusions and Future Directions

The term “composite” denotes materials made of two or more components (organic or inorganic) with substantially different physical or chemical properties. On the other hand, the term “nanocomposite” is applied once the size of one of the composite’s structural constituents is in the range of 1 to 100 nm. Over the years, biocompatible nanocomposites have been found to be beneficial substances for medical applications, such as the treatment of postoperative adhesions. Compared to conventional types of materials (e.g., biopolymers), nanocomposites offer extraordinary advantages for use in postoperative adhesions, including excellent biocompatibility and enhanced mechanical characteristics. A wide range of nanosized organic and inorganic substances can be mixed to generate diverse nanocomposites with distinct physicochemical, physical, and biological properties; this provides opportunities for the management of various adhesions occurring in different parts of the body. According to the literature, the most developed and used nanocomposites are indeed organic-organic composites (nanostructured polymeric constructs). Hence, different types of organic-inorganic nanocomposites can be explored for their potential to reduce and prevent postsurgical adhesions and accelerate tissue healing. For instance, the carbon family (e.g., carbon nanotubes (CNTs)) may be considered a part of novel formulations of nanocomposites due to their excellent characteristics, such as superior mechanical properties, tissue regeneration, and drug delivery. Still, the toxicity and high manufacturing cost of this kind of material should be considered when developing nanocomposites based on carbon family members. Apart from their composition, newly developed nanocomposites should have the ability to modulate the biological phenomena involved in postoperative adhesions in favor of improved tissue healing and repair, such as coagulation, inflammation, fibrinolysis, and angiogenesis. Moreover, other characteristics of nano-engineered biomaterials and scaffolds can affect the rate of tissue healing in damaged sites. This includes applied nanocomposites’s hydrophilicity and their ability to exchange body fluids, as well as their wound generation potential.

Current research has indicated that nanocomposites can be formulated and fabricated as smart scaffolds (e.g., hydrogels) to release therapeutic cargoes in response to various stimuli (e.g., changes in pH level). This provides a desirable condition for injury management with minimal side effects on the repair and regeneration of damaged tissues. In this sense, biocompatible nanocomposite scaffolds made of stimuli-responsive polymers (e.g., polypyrrole (PPy)) can be beneficial for the acceleration of tissue healing and adhesion management. Future research may focus on the fabrication and utilization of 3D-printed constructs by using nanocomposites as raw materials. These constructs can be defined as the next generation of antiadhesive constructs in the concept of precision medicine. Ultimately, the cost of nanocomposite products should be considered an important factor to compete with the available products in the market.

## Figures and Tables

**Table 1:** A summary of proposed and applied treatment approaches for the management of postoperative adhesions.

Treatment Approach	Components	Hallmarks	Refs.
Solid films and membranes	PCL nanofibrous mat modified with chitosan	- Inhibited adhesion and proliferation of fibroblasts - Promoted endothelial cell performance in terms of adhesion and proliferation, hence serving as an antiadhesion barrier - Antibacterial activity	[69]
	CMC/ORC composite gauze	- Hemostatic and antibacterial activity - Prevented the adhesion of fibroblasts - Efficiently inhibited adhesion formation	[70]
	PLA-PEG-PLA film	- Decreased peritoneal adhesion in rats - Better performance than Seprafilm^®^	[71]
Liquid hydrogels	Poly-L-lysine with PEG	- Decreased PA in rats - Inhibited cell–tissue adhesions - Altered the fibrin formation process in vitro	[72,73]
	Fibrin glue	- Decreased adhesion in different species (rats, rabbits, and humans) while maintaining a normal peritoneal healing process	[74]
Anticoagulant agents	Heparin and derivatives	- Accelerated thrombin inactivation, anticoagulation, effective - anti-PA properties without impaired peritoneal healing process	[75,76]
	Thromboxane A2 receptor blockers (Ridogrel)	- Prevented platelet activation - Exerted anti-PA activity in a rabbit model	[77]
Anti-inflammatory agents	Vitamin E	- Decreased PA via oral or IP administration in rats	[78,79]
	DEX	- Decreased PA after IV injection through antioxidant and anti-inflammatory effects in rats	[80]
Fibrinolytic agents	Octreotide	- Demonstrated anti-PA properties after IP or IM injection in mice - Led to overexpression of tPA and downregulated expression of TGF-β1 and VEGF	[81]
	Ghrelin	- Showed anti-PA activity in mice - Blocked TGF-β signaling pathway	[82]
Combination therapy	Heparin/HA combination	- Declined absorption of heparin in rats and promoted safety	[76]
	Cefoxitin sodium-loaded PLGA electrospun nanofibrous membranes	- Reduced PA in rat animal model	[83]

**Abbreviations:** CMC, carboxymethyl cellulose; DEX, dexmedetomidine; IM, intramuscular; IP, intraperitoneal; IV, intravenous; PA, peritoneal adhesion; PEG, poly(ethylene glycol); PCL, poly(caprolactone); PLA, poly(lactic acid); PLGA, poly(lactic-co-glycolic acid); ORC, oxidized regenerated cellulose; TGF-β, transforming growth factor-beta; VEGF, vascular endothelial growth factor.

**Table 2:** Pros and cons of polymers utilized to prevent postoperative adhesions.

Polymer	Advantages	Disadvantages	Refs.
Natural polymers			
Alginate	- Water-soluble - Good biocompatibility - Controllable mechanical properties through non-toxic crosslinking reactions	- Limited control of mechanical properties - Ion leaching leads to instability	[86,87,88]
Hyaluronic acid (HA)	- Good biocompatibility and swelling properties - Controllable mechanical properties by crosslinking reactions	- Rapid biodegradation has made it necessary to use it in composites with other biomaterials	[89,90,91,92]
Gelatin	- Good biocompatibility - Controllable mechanical properties by crosslinking reactions	- Toxic side effects due to crosslink residuals (e.g., glutaraldehyde) - Fast degradation rates	[93,94]
Chitosan	- Good biocompatibility and biodegradability - Hemostatic activity - Antibacterial properties - Free-radical-scavenging performance	- Possible risk to patients allergic to chitin	[95,96]
Carboxymethylcellulose (CMC)	- Good biocompatibility and biodegradability - Proper hydrophilicity - Bioresorbability within a few days	- Poor mechanical properties	[94,97,98]
Synthetic polymers			
Polylactic acid (PLA)	- Good biodegradability - High melting point - Perfect strength	- Poor processability - Poor flexibility - High cost - Requires sutures for fixation of PLA-based barriers	[99]
Polycaprolactone (PCL)	- Good mechanical properties (i.e., tensile strength, elongation, and impact strength) - Good biodegradability	- Highly hydrophobic - Low flexibility - Requires sutures for fixation of PLA-based barriers - Prolonged biodegradability	[100,101]
Polyvinyl alcohol (PVA)	- Good biocompatibility - Water-soluble - Good physical properties - Low cost	- High swelling and dissolving properties - Toxic side effects regarding crosslinking agents (e.g., glutaraldehyde)	[102,103,104]
Polyethylene glycol (PEG)	- Good biocompatibility - Inhibited the growth of biofilm	- Fast degradation in the body (less than 7 days)	[105]

**Table 3:** A summary of commercially available antiadhesive products in clinical administration or development [17].

Antiadhesive Formulation	Product Name	Used Material
Hydrogel	Guardix-sol^®^	Sodium hyaluronate/carboxymethyl cellulose (CMC)
	Hyalobarrier^®^	Hyaluronic acid (HA)
	Hyskon^®^	Dextran
	COVA™	Collagen
	Oxiplex^®^	Polyethylene glycol (PEG)/CMC
	A-part Gel^®^	CMC/polyvinyl alcohol (PVA)
	Guardix-SG^®^	Alginate/Poloxamer
	Medishield™	CMC/polyethylene
	NOCC™ (Kytogenics)	N, O-carboxymethyl chitosan hydrophilic
	Oxyplex^®^	CMC and polyethylene oxide
Film	Surgicel^®^	Oxidized regenerated cellulose (ORC)
	Interceed^®^	Oxidized regenerated cellulose (OCR)
	Seprafilm^®^ (CMC)	Modified HA and CMC
	Incert^®^	HA
	Carbylan™-SX	HA
	REPEL-CV^®^	PEG/polylactic acid (PLA)
	SurgiWrap^®^	PLA
	Gore^®^Preclude^®^	Polytetrafluoroethylene
Sponge powder	DuraGen^®^ Plus	Ultrapure collagen
	Sepraspray™	Sodium hyaluronate/CMC
	4DryField^®^	Purified natural starch
Solution	Sepragel^®^	Sodium hyaluronate/CMC
	Sepracoat^®^ 0.04%	HA-phosphate-buffered saline
	ACP^®^ gel	HA
	Lubricoat^®^	HA
	Intergel^®^	HA
	Adept^®^	Icodextrin
	Hyskon^®^ (Dextran 70)	Dextrin solution
	Adcon-P^®^	Gelatin and proteoglycan solution
	Guardix-SL^®^	Liquid-based HA and CMC solution
Spray	AdSpray^®^	N-hydroxysuccinimide-modified, carboxymethyl dextrin, and trehalose
	Sprayshield™	Water, PEG, lysine
	SprayGel^®^	PEG

## Data Availability

No new data was created.
